# Force-Controlled Biomechanical Simulation of Orthodontic Tooth Movement with Torque Archwires Using HOSEA (Hexapod for Orthodontic Simulation, Evaluation and Analysis)

**DOI:** 10.3390/bioengineering10091055

**Published:** 2023-09-07

**Authors:** Ellen Haas, Andreas Schmid, Thomas Stocker, Andrea Wichelhaus, Hisham Sabbagh

**Affiliations:** Department of Orthodontics and Dentofacial Orthopedics, LMU University Hospital, LMU Munich, Goethestraße 70, 80336 Munich, Germany; ellen.haas@med.uni-muenchen.de (E.H.); schmid.92@gmx.de (A.S.); th.stocker@med.uni-muenchen.de (T.S.); kfo.sekretariat@med.uni-muenchen.de (A.W.)

**Keywords:** orthodontics, 3D measurement, force control, biomechanics, hexapod

## Abstract

This study aimed to investigate the dynamic behavior of different torque archwires for fixed orthodontic treatment using an automated, force-controlled biomechanical simulation system. A novel biomechanical simulation system (HOSEA) was used to simulate dynamic tooth movements and measure torque expression of four different archwire groups: 0.017″ x 0.025″ torque segmented archwires (TSA) with 30° torque bending, 0.018″ x 0.025″ TSA with 45° torque bending, 0.017″ x 0.025″ stainless steel (SS) archwires with 30° torque bending and 0.018″ x 0.025″ SS with 30° torque bending (*n* = 10/group) used with 0.022″ self-ligating brackets. The Kruskal–Wallis test was used for statistical analysis (*p* < 0.050). The 0.018″ x 0.025″ SS archwires produced the highest initial rotational torque moment (M_y_) of −9.835 Nmm. The reduction in rotational moment per degree (M_y_/R_y_) was significantly lower for TSA compared to SS archwires (*p* < 0.001). TSA 0.018″ x 0.025″ was the only group in which all archwires induced a min. 10° rotation in the simulation. Collateral forces and moments, especially F_x_, F_z_ and M_x_, occurred during torque application. The measured forces and moments were within a suitable range for the application of palatal root torque to incisors for the 0.018″ x 0.025″ archwires. The 0.018″ x 0.025″ TSA reliably achieved at least 10° incisal rotation without reactivation.

## 1. Introduction

In orthodontic therapy with fixed appliances, biological tooth movement is achieved by applying forces and moments to the teeth and further to the surrounding structures involved—the periodontal ligament and alveolar bone [1,2,3]. For efficient tooth movement and avoidance of adverse effects such as pain, extensive hyalinization and root resorption, knowledge and control of orthodontically applied forces and moment magnitudes is required [4,5]. 

While force magnitudes can be accurately determined for some components used with fixed appliances, such as elastic chains, nickel–titanium springs, cantilevers, and intermaxillary elastics, the resulting forces of more complex archwire bends are difficult to determine or even estimate in vivo [6]. However, this knowledge is particularly important for critical orthodontic tooth movements frequently leading to apical root resorption, such as orthodontic torque application [7]. Clinically, the application of palatal root torque is indicated for orthodontically correct incisor inclination or to maintain it, e.g., during incisor retraction [7]. To obtain information about forces, moments, movements or similar, two methods of in vitro investigations are established in orthodontic science: digital, finite element (FE) simulation and biomechanical experiments [8,9,10].

FE simulations are based on computer-aided numerical methods and can perform analyses on a virtual model [11,12]. However, FE simulations have limitations implementing complex structures consisting of multiple components and reflect dynamic material interactions [13]. Simplifications are necessary during the modeling process, thus the clinical implications of FE simulations are to be interpreted carefully and may require experimental verification in more complex cases [14].

Biomechanical experiments, on the other hand, can serve to investigate material characteristics, such as geometrical accuracy, Young’s modulus, surface properties and frictional behavior, among others [15,16,17]. Biomechanical simulation systems can additionally address complex dynamic physical behaviors and material interactions during simulated clinical procedures. Most biomechanical simulation systems focus mainly on specific individual tooth movements, such as distalization or mesialization [18] or static torque expression and rotating movements [15,19]. Only a few experimental setups are designed to simulate complex motion sequences, such as dynamic torque expression [20,21].

HOSEA is a novel biomechanical simulation system based on a hexapod platform with a parallel kinematics positioning system that allows coordinated multi-axis motion in all six degrees of freedom. Controlled by a force–moment-dependent algorithm, this platform can move autonomously with respect to defined points such as the center of force or center of resistance to simulate the dynamic behavior of orthodontic mechanics.

The aim of this study was to investigate the dynamic torque expression of different orthodontic torque archwires during tooth movement using an automated, force-controlled biomechanical simulation system.

## 2. Materials and Methods

### 2.1. Biomechanical Simulation System

**Figure 1:**
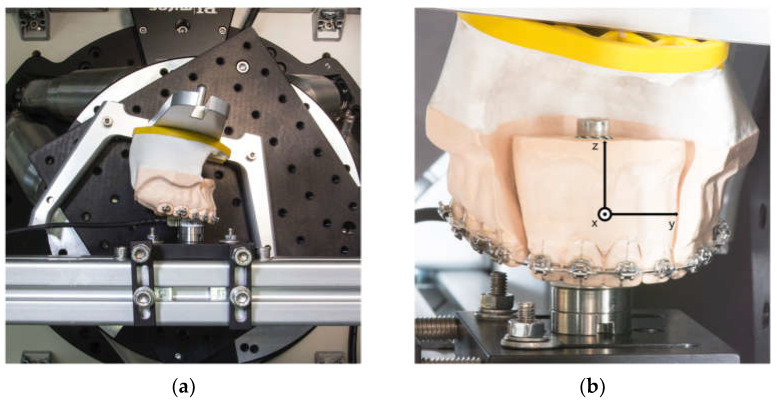
(**a**) Plaster model in the examination chamber of HOSEA; the force-torque sensor is attached to the anterior segment, while the posterior segment is connected to the moving Stewart platform. (**b**) Plaster model in the starting position, with superimposed coordinate system used for the measurements.

A hexapod or Stewart platform HP-550 (PI GmbH, Karlsruhe, Germany) was used as central motion element of HOSEA. The integrated six linear actuators are able to move a platform in six degrees of freedom (three translational and three rotational) with the highest precision and almost play-free. A computer and a control unit (Geobrink LV 8-axis, Delta Tau Data Systems Inc., Chatsworth, LA, USA) were used to control the Stewart platform. The control software was developed using LabView 12 (NI, Austin, TX, USA). As the main part of the software, an algorithm was implemented to allow force-controlled movement. This enables the platform to move autonomously, depending on the continuously measured forces and moments. A six-axis force-torque sensor was integrated into HOSEA to measure forces and moments (Nano17 SI-50-0.5, ATI Industrial Automation, New York, NY, USA). It is able to measure a force range of 70 N in the axial direction and 50 N for the two space vectors at a resolution of 1/80 N. The measuring range for the torsional moment is 500 Nmm in all spatial planes at a resolution of 1/16 Nmm (Figure 1a).

All experiments were conducted at ϑ = (36 ± 1) °C in a temperature chamber with a temperature controller (TOHO TM-105, TOHO electronics, Sagamihara, Japan), because of the temperature-dependent material characteristics of the nickel–titanium component of the investigated archwires. HOSEA as a whole was housed in this chamber.

An experimental plaster model was made based on a typodont (Frasaco GmbH, Tettnang, Germany). Simulating an extraction therapy case, the first premolars were removed and replaced by canines. The model was divided into two segments, an anterior (teeth 12, 11, 21, 22) and one posterior segment (teeth 13–17, 23–27). The anterior segment was attached to the sensor and the posterior segment to the Stewart platform by a SAM articulator plate and base (SAM Praezisionstechnik GmbH, Munich, Germany) (Figure 1). The sensor and the anterior segment are fixed onto a strut of the housing, while the posterior segment is moved by the hexapod. The interbracket distance between the first molar and the lateral incisor was set at 28 mm on both sides.

Subsequently, the experimental model and four plastic incisors (ANA-4 ZP, Frasaco GmbH, Tettnang, Germany) were digitized using a desktop 3D scanner (Everest Scan, KaVo Dental GmbH, Biberach an der Riß, Germany). The scans of the incisors were post-processed with modeling software (Autodesk Meshmixer Version 3.5, Autodesk Inc., San Rafael, CA, USA). The length of the incisors’ roots were set according to average length values [22].

The model scan and the tooth scans were matched with MeshLab [23] using an iterative closest point algorithm. Afterwards, MeshLab was utilized to determine the root surface barycenter which was identified as the center of resistance of the anterior segment of the four incisors. This center of resistance was set as the pivot point of HOSEA. Additionally, root surfaces were used to calculate the coefficients of a transformational matrix to specify the movement for each spatial direction. To describe directions of forces and moments, a coordinate system was defined on the anterior segment of the teeth (Figure 1b). It is aligned in such a way that its origin matches the center of the sensor. The position has been designed to coincide with the position of the calculated center of resistance. The sensor has a coordinate system determined by the manufacturer. The manufacturer’s coordinate system was adapted to the anterior tooth coordinate system by employing mathematical transformation. As a result, the data measured by the sensor can be described in anterior segment system coordinates. Vertical movements are defined in the coordinate system along the *z*-axis, while anteroposterior movements are defined along the *x*-axis. The distance between the sensor center and the center of force of the anterior tooth segment was considered mathematically.

Self-ligating orthodontic brackets (0.022″, In-Ovation R, Dentsply Sirona, New York, NY, USA) were passively bonded to the plaster model using a 0.021″ x 0.025″ stainless steel archwire aligned along the marked FACC points [24].

The starting position of the mounted model in the experimental setup was determined with the help of the previously utilized 0.021″ x 0.025″ stainless steel archwire to obtain a fully passive fit. It is important to point out that in the given experimental setup, the position of the anterior segment is fixed while the posterior jaw segment is moved by the HOSEA system.

### 2.2. Measurements and Biomechanical Simulation of Tooth Movement

For the measurements and biomechanical simulations, pre-torqued archwires were ligated to the jaw model after passive alignment and initial calibration with supplementary steel ligatures (Forestadent GmbH, Pforzheim, Germany) in the starting position of the experimental setup of HOSEA.

In total, 40 torque archwires were examined. Four groups of ten samples were bent and adapted to the model by the same experienced clinician (Table 1). The measurement cycle was finished as soon as the anterior segment stopped rotating. All experiments were conducted at the lowest velocity to reduce the effect of the movements on the measurements.

### 2.3. Statistical Analysis

Graphs were generated using OriginPro 2020b (OriginLab Corporation, Northampton, MA, USA), and tables were prepared using Microsoft Excel 2016 (Microsoft Corporation, Redmond, WA, USA).

For statistical analysis, Kruskal–Wallis test with a significant value of α = 0.050 was conducted in IBM SPSS Statistics 26 (International Business Machines Corporation, Armonk, NY, USA).

## 3. Results

**Figure 2:**
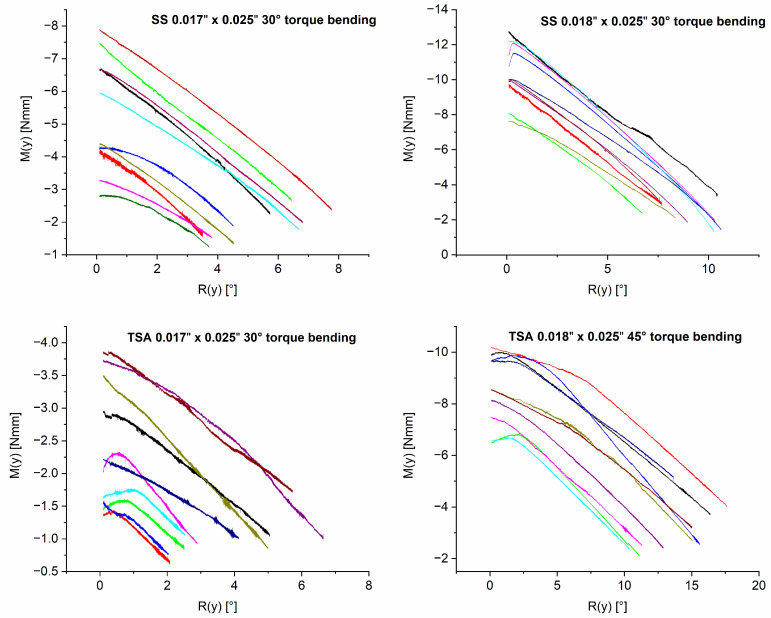
Graphs depicting rotational moments (Nmm) at the center of force in relation to the rotation of the segment (°). All measurements started at a rotational angle of 0° with an initial moment M_y_ (R_y_ = 0.1°). HOSEA rotates the posterior segment until My has come to an equilibrium position and rotation has stopped. Every colored curve corresponds to the measurement of one archwire. Each graph represents the archwires within a sample group.

Figure 2 illustrates the rotational moment M_y_ in relation to the rotation of the anterior segment R_y_. Each of the four graphs represents a single archwire category, in which the 10 archwires are individually identified by different colors. The results of the measurements are presented according to the implemented Tweed coordinate system for the anterior tooth segment (Figure 1b). Since forces and moments were calculated at the center of resistance of the segment, the resulting force and moment values’ direction indicate specific tooth movements. Negative values of M_y_ resemble the rotational moment resulting in a palatal root torque expression. Initial moments and forces were measured at 0.1° of rotation to reduce initial irregularities. Figure 2 and Table 2 show that larger wire dimensions account for higher rotational moments, initially and throughout the rotation, compared to smaller dimensions. The SS archwires have a tendency towards higher rotational moments. The highest rotational moment was observed in the SS 0.018″ x 0.025″ group, measuring −9.835 Nmm at 1° rotation on average.

The four graphs (Figure 2) also illustrate a difference in rotation achievable through archwires within each group. To investigate this further, Table 2 differentiates between certain degrees of rotation (2.5°, 5° and 10°), the according rotational moment My and the number of archwires successfully achieving rotation to the respective degree. Within a group, some samples failed to rotate to a certain degree. All investigated 0.018″ x 0.025″ TSA archwires successfully managed to rotate the segment for 10°. In contrast, in the 0.018″ x 0.025″ SS group, only four archwires reached this amount of rotation. The 0.017″ x 0.025″ SS and TSA archwires failed to reach this mark. Only a reduced number of 0.017″ x 0.025″ archwires caused a 5° rotation. Within the TSA 0.017″ x 0.025″ group, eight archwires achieved a 2.5° rotation (Table 2).

In general, all four types of archwires had a reduction in the rotational moment as soon as the anterior segment rotates (Figure 2). However, the rate of rotational moment decrease varied depending on the archwire group. SS archwires showed a significantly higher moment reduction per degree (M_y_/R_y_) than TSA archwires (Table 3). Statistical analysis revealed a difference between the two material classifications but not between the archwire sizes (*p* = 0.006 for 0.017″ x 0.025″ wire dimensions, *p* < 0.001 for 0.018″ x 0.025”). Furthermore, the rotational moment depletion rate of the TSA groups showed a smaller standard deviation than the SS groups (Table 3).

During rotation, collateral forces and moments were observed. The initial values of these forces and moments at 1° rotation are reported in Table 4. The 0.018″ x 0.025″ SS group showed the highest values for the collateral force F_y_ of −0.788 N. Statistical analysis showed that the two SS archwire groups differed significantly (*p* = 0.042) (Table 4). They were also both different from the 0.017″ x 0.025″ (30° torque) TSA group (*p* = 0.037 for 0.017″ x 0.025″, *p* = 0.000 for 0.018″ x 0.025″). A significant difference (*p* = 0.010) could also be observed among the TSA groups. There was no significant difference between the SS 0.018″ x 0.025″ (30° torque) and TSA 0.018″ x 0.025″ (45° torque) groups (*p* = 0.137). Focusing on the extrusive force F_z_, larger values could be observed in the 0.018″ x 0.025″ archwire groups (Table 4). A significant difference was observed between the 0.017″ x 0.025″ and 0.018″ x 0.025″ archwire dimensions in each material group (*p* = 0.003 for SS, *p* = 0.001 for TSA). The SS group showed higher mean values and standard deviations compared to the two TSA groups (Table 4). M_x_ corresponds to a rotational movement that leads to extrusion on one side of the incisal segment and intrusion on the other.

**Figure 3:**
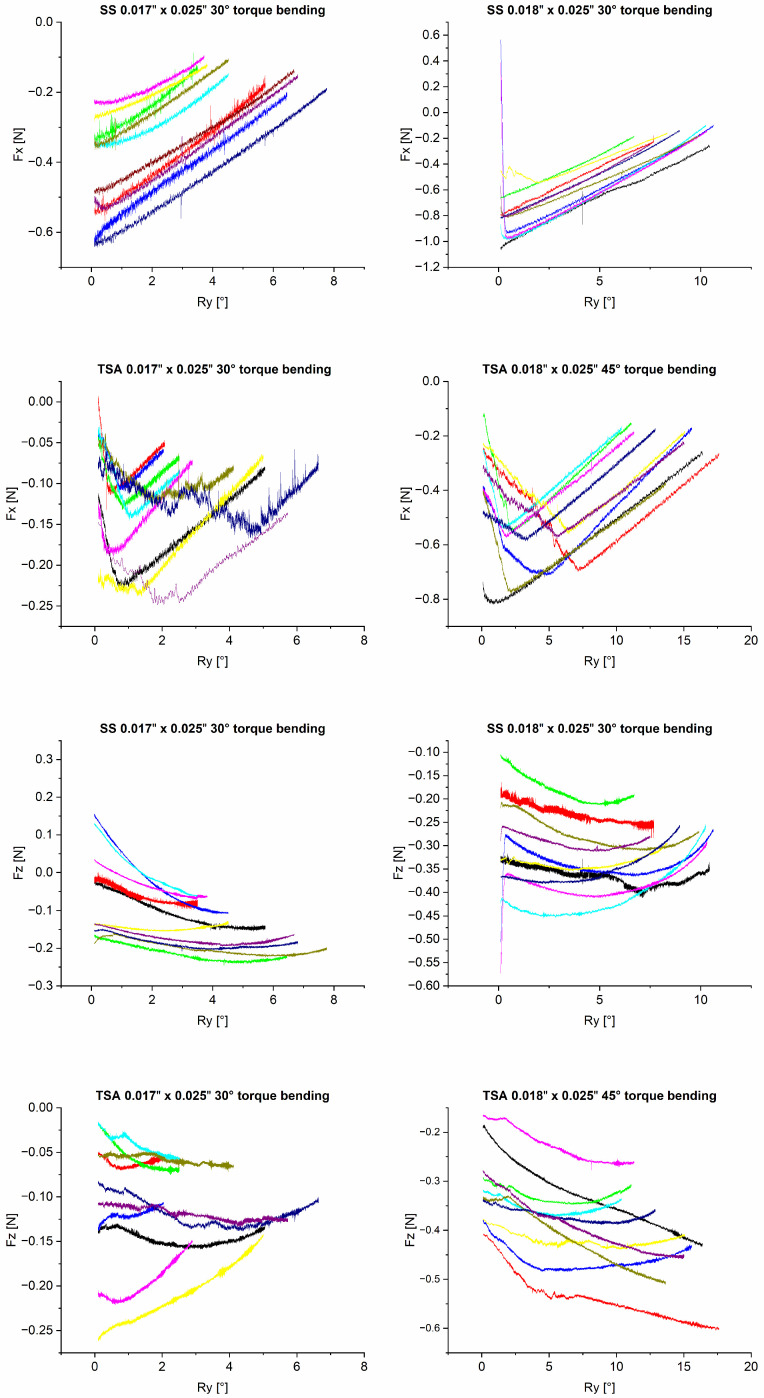
Graphs depicting collateral forces F_x_ and F_z_ at the center of force in relation to the rotation of the segment (°). All measurements started at a rotational angle of 0° with an initial moment My (R_y_ = 0.1°). Every colored curve corresponds to the measurement of one archwire. Each graph represents the archwires within a sample group.

Figure 3 depicts the measured collateral forces F_x,_ representing a retractive force, and F_z_, corresponding to an extrusive force. Focusing on F_x,_ both SS groups showed a linear force reduction during the rotation of the anterior segment. In contrast, in the TSA groups, the extrusive force increased at the beginning of the rotation, peaked during the course of movement and then decreased. The measured retractive Force F_x_ ranged from a minimum of 0 N to a maximum of −1.0 N and was lowest in the TSA 0.017″ x 0.025″ group.

The measured extrusive force F_z_ was generally larger in the 0.018″ x 0.025″ archwire groups. For SS 0.018″ x 0.025″ archwires, extrusive force F_z_ values ranged between −0.1 N and −0.45 N. For TSA 0.018″ x 0.025″ archwires, F_z_ values ranged between −0.15 N and −0.6 N. In comparison, measured forces for the SS 0.017″ x 0.025″ archwire group ranged between 0.1 N and −0.2 N, while the extrusive force of TSA remained in the negative section between 0 N and −0.3 N.

In general, large variations in F_x_ and F_z_ between archwires within all groups were evident.

## 4. Discussion

The dynamic course of torque expression of different orthodontic torque archwires was investigated using the fully automated and force-controlled biomechanical simulation system HOSEA.

Clinically, the application of rotational moments between 5 and 20 Nmm has been recommended to achieve adequate torque on the incisors [25,26]. In this study, mean values for rotational moments (M_y_) ranged between 2.284 Nmm and 9.835 Nmm, where none of the samples exceeded the upper limit of 20 Nmm. This is in line with the lower range of comparable torque measurements in the literature [15,21,27]. Based on the archwire–bracket configurations used, higher values would have been expected. Theoretically, the torsional play between bracket and archwire can be calculated through geometric considerations [28]. However, the manufacturer’s specifications for the dimensions of both, bracket as well as archwire, often do not correspond to the actual sizes due to manufacturing tolerances. Studies have shown that bracket slots may be oversized by up to 24% in some areas [29]. Additionally, archwire examinations have shown that archwire sizes are outside lower tolerance limits given by the relevant normative standards [30]. In this context, the results of the present study suggest that more torsional play effectively occurs than theoretically anticipated [31,32], leading to a significant loss of rotational moment.

As can be seen in Figure 2, the torsional moments from the TSA samples with the smaller dimensions are very low, leading to the conclusion that the chosen configuration of 0.017″ x 0.025″ with 30° of torque in combination with a 0.022″ slot is not suitable to perform incisor torque movements. Therefore, the sample group based on this combination will not be considered further in this discussion.

In addition to the magnitude of the applied rotational moments, the rotational moment reduction rate (M_y_/R_y_) is of particular clinical importance. In this regard, significant differences were found between the groups investigated. The present study compared SS with TSA archwires, in which the posterior segment was made of stainless steel and the anterior segment was made of a superelastic nickel–titanium alloy. Compared to stainless steel alloys, nickel–titanium alloys exhibit a low Young’s Modulus and show constant force-deflection plateaus over rather long deflection ranges [33,34]. This was also reflected in the results of this study, as the TSA groups showed a lower rotational moment depletion rate (around 0.4 Nmm/°) compared to the SS groups (between 0.7 and 0.9 Nmm/°).

Furthermore, the need for reactivations of treatment mechanics is clinically relevant. Reactivation becomes necessary when suitable moments are no longer exerted by the orthodontic appliance. In this study, some specimens failed to achieve a reasonable amount of rotation during the simulations, while the 0.018″ x 0.025″ TSA was the most reliable in achieving a rotation of at least 10°. On the other side, depending on the magnitude of initial starting moment M_y_, the achievable rotational angle may also range up to 17.5° under the same experimental conditions. It was shown that the final rotational angle depends on the initial rotational moment M_y_ and the moment depletion rate. Clinically, this fact may be of concern, especially because a similar wide spread of final rotational angles was also found in all sample groups. One possible explanation for the wide range of initial rotational moments M_y_ can be found in the manual torque bending and measuring procedure, which is performed through a visual template comparison. Even though all archwires were bent by the same experienced clinician, visual inspection cannot exclude slight variations in torque bends and bends to adjust the archwire shape to the template. Although the anterior segments in the TSA group were pre-torqued, these archwires were also adapted to the shape of the template and a comparable pattern was found here as well. This probably also explains the wide variation in the measured extrusive and retractive collateral forces in all archwire groups investigated. A calibrated measuring device for the effectively applied torque angle is under development and will be validated for further research.

In addition to the measured rotational moment M_y_ resulting in palatal torque movement of the incisors, significant collateral forces and moments were observed in F_z_, F_x_ and M_x_. This corresponds to an extrusion, as well as a mesio-distal movement and a rotation around the *y*-axis. Extrusion and retraction of the anterior segment must generally be anticipated biomechanically during torque application [7,35]. The sideways movement and rotation are more likely to result from asymmetrical adjustment of the archwires. This effect may be more pronounced in contrast to the clinical situation, as contacts between bracket and archwire cannot be released by chewing forces and, in absence of lubricating saliva, more pronounced frictional phenomena may occur. The measured collateral moment M_x_ was higher for the SS groups with values of 2.099 Nmm and 2.104 Nmm compared to the TSA groups with values ranging between −0.586 Nmm and 0.051 Nmm. The difference between the groups supports the assumptions, since TSAs are prefabricated in the anterior segment and thus show lower asymmetry In comparison with the manually bent SS archwires.

In vitro investigations with biomechanical test devices such as HOSEA are limited to purely mechanical simulations and cannot reproduce orthodontic tooth movements as a biological process. In addition to the absence of a periodontal ligament (PDL) and saliva, simplifying assumptions were made in the simulations by using a standardized model and in defining a static center of resistance of the anterior segment. Although the setup used cannot simulate a PDL, the software allows the integration of previously calculated characteristics, such as the center of resistance and the root resistance, into the simulated movement. Despite the fact that, at present, it is recognized that each tooth has a unique center of resistance, in this study, in order to simulate the movement of the anterior block of teeth, a joint center of resistance was calculated as proposed in previous studies [36,37]. It was determined on the basis of indications in the literature at 10.324 mm apically of the combined center of force of the four incisors [2,37,38].

Compared to in silico FE analysis, HOSEA allows the observation and analysis of the biomechanical properties of physical orthodontic appliances, whereas FE analyses are solely based on calculative models [3,39]. FE simulations calculate forces and moments generated by mechanically idealized orthodontic appliances or archwires. The present investigations can thus be perceived as an addition and validation to FE studies by providing values for respective computations and allowing comparisons between investigation methods. The presented setup can perform force-controlled simulations of different tooth movements in terms of dynamic three-dimensional measurements, which is rare in orthodontic research to date [10,20,40]. Furthermore, it is possible to conduct these measurements on real patient’s dental casts in order to validate treatment plans in highly complex cases.

Considering the results and limitations of this study, the following clinical implications can be derived:

- The 0.017″ x 0.025″ archwires in combination with 0.022″ slot brackets produced forces and moments that were too low to achieve adequate palatal incisor root torque, regardless of material group. Therefore, the use of 0.018″ x 0.025″ archwires with 30° torque bends (SS) or 45° torque bends (TSA) are recommended for clinical use.
- Although higher initial moments (M_y_) were measured for the 0.018″ x 0.025″ SS archwires, the 0.018″ x 0.025″ TSA archwires exhibited a lower moment reduction rate, indicating a reduced need for reactivation and appearing to be more suitable for applying more constant rotational moments. Due to collateral effects such as extrusive forces occurring during torque expression, the application of compensatory vertical bends should be considered.

## 5. Conclusions

The dynamic course of torque expression of SS and TSA archwires during force-controlled simulation of orthodontic tooth movement was investigated using the novel biomechanical test stand HOSEA. Both SS and TSA archwires produced suitable moment magnitudes for palatal root movements at 0.018″ x 0.025″ dimensions in combination with 0.022″ slot brackets. In contrast, 0.017″ x 0.025″ archwires did not produce sufficient moment magnitudes and thus did not achieve adequate palatal root torque regardless of the material. TSA archwires showed significantly lower rotational moment reduction rate over time in comparison with SS archwires.

## Figures and Tables

**Table 1:** Sample groups used for the investigations, their material composition, the archwire dimensions and the amount of applied torque bends.

Archwire Material	Material Identification	Applied Torque	Sample Size	Archwire Size
Stainless steel	X10CrNi 18-8	30°	10	0.017″ x 0.025″
Stainless steel	X10CrNi 18-8	30°	10	0.018″ x 0.025″
Torque-segmented archwire	X5CrNi 18-10 Nickel Titanium	30°	10	0.017″ x 0.025″
Torque-segmented archwire	X5CrNi 18-10	45°	10	0.018″ x 0.025″
	Nickel Titanium			

**Table 2:** Mean rotational moments at the center of force at different stages of rotation for the different archwires investigated. Each archwire category consisted of 10 archwires. The number of archwire samples per group succeeding to rotate the anterior segment by a certain degree (1°, 2.5°, 5°, 10°) are shown in the line “No. of samples”. Only the successful samples were included in the calculation at the defined rotational position of the anterior segment. If the amount of rotation was not been reached by any of the archwire samples in the respective group this is indicated by “-“.

Archwire Category	Torque Bending	M_y_ (Nmm) (SD)	M_y_ (Nmm) (SD)	M_y_ (Nmm) (SD)	M_y_ (Nmm) (SD)
		R_y_ = 1°	R_y_ = 2.5°	R_y_ = 5°	R_y_ = 10°
0.018″ x 0.025″ TSA No. of samples	45°	8.468 (1.330) 10	−8.207 (1.372) 10	−7.297 (1.576) 10	−3.927 (3.753) 10
0.018″ x 0.025″ SS No. of samples	30°	−9.835 (1.701) 10	−8.604 (1.563) 10	−6.393 (1.414) 10	−2.579 (0.916) 4
0.017″ x 0.025″ SS No. of samples	30°	−4.914 (1.648) 10	−4.004 (1.547) 10	−3.571 (0.664) 5	-
0.017″ x 0.025″ TSA No. of samples	30°	−2.284 (0.906) 10	−1.903 (0.879) 8	0.022 (0.022) 3	-

**Table 3:** Rotational moment depletion rate for the different archwire categories. The sample size for each archwire category was *n* = 10.

Archwire Category	Torque Bending	M_y_/R_y_ (Nmm/°)	SD	*p*
0.018″ x 0.025″ TSA	45°	−0.405	0.045	<0.001
0.018″ x 0.025″ SS	30°	−0.898	0.108	
0.017″ x 0.025″ TSA	30°	−0.396	0.083	0.006
0.017″ x 0.025″ SS	30°	−0.717	0.136	

**Table 4:** Average initial forces and moments for the different archwire categories in and around the three spatial dimensions (x, y, z). The superscripts correspond to a significant difference between the measured variable and the examined sample of the corresponding archwire categories titled variables ^(a, b, c, d^) according to the Kruskal–Wallis test with α = 0.050. The sample size of each archwire category was *n* = 10.

	Archwire Category	Torque Bending	F_x_ (N) (SD)	F_y_ (N) (SD)	F_z_ (N) (SD)	M_x_ (Nmm) (SD)	M_y_ (Nmm) (SD)	M_z_ (Nmm) (SD)
a	0.017″ x 0.025″ SS	30°	−0.399 (0.133) ^bc^	−0.195 (0.189) ^cd^	−0.083 (0.092) ^bd^	2.099 (2.037) ^c^	−4.914 (1.648) ^b^	0.018 (0.086) ^d^
b	0.018″ x 0.025″ SS	30°	−0.788 (0.159) ^ac^	−0.193 (0.260)	−0.300 (0.089) ^a^	2.104 (2.723)	−9.835 (1.701) ^ac^	−0.079 (0.173)
c	0.017″ x 0.025″ TSA	30°	−0.150 (0.055) ^abd^	0.070 (0.100) ^ad^	−0.113 (0.069) ^abd^	−0.586 (1.189) ^a^	−2.284 (0.906) ^bd^	−0.070 (0.194)
d	0.018″ x 0.025″ TSA	45°	−0.457 (0.160) ^c^	0.078 (0.156) ^ac^	−0.325 (0.081) ^ac^	0.051 (1.664)	−8.468 (1.330) ^c^	−0.886 (0.881) ^a^

## Data Availability

Data are available on request from the corresponding author.
